# Are superficial neuromasts proprioceptors underlying fast copulatory behavior?

**DOI:** 10.3389/fncir.2022.921568

**Published:** 2022-08-23

**Authors:** Noraida Martinez-Rivera, Jose L. Serrano-Velez, Irma I. Torres-Vazquez, R. Brian Langerhans, Eduardo Rosa-Molinar

**Affiliations:** ^1^Biological Imaging Group, Department of Pharmacology and Toxicology, The University of Kansas, Lawrence, KS, United States; ^2^Biology Department, University of Puerto Rico-Rio Piedras, San Juan, Puerto Rico; ^3^Puerto Rico Center for Environmental Neuroscience, Institute of Neurobiology, University of Puerto Rico-Medical Sciences, Old San Juan, Puerto Rico; ^4^Bi-campus Neuroscience Graduate Program, The University of Kansas, Lawrence, KS, United States; ^5^Department of Biological Sciences, North Carolina State University, Raleigh, NC, United States

**Keywords:** unpaired anal fin, superficial neuromasts, proprioceptor, fast copulatory behavior, gonopodium, mosquitofish, mechanosensory receptor, 3D kinematics

## Abstract

In male Poeciliid fishes, the modified anal fin (i.e., gonopodium) and its axial and appendicular support are repositioned within the axial skeleton, creating a novel sexually dimorphic ano-urogenital region. During copulation, the relative location of the gonopodium is crucial for successful insemination. Therefore, the repositioning of these structures and organ relied on the reorganization of the efferent circuitry that controls spinal motor neurons innervating appendicular muscles critical for the movement of the gonopodium, including the fast and synchronous torque-trust motion during insemination attempts. Copulation occurs when a male positions himself largely outside a female’s field of view, circumducts his gonopodium, and performs a rapid, complex maneuver to properly contact the female urogenital sinus with the distal tip of the gonopodium and transfers sperm. Although understanding of the efferent circuitry has significantly increased in the last 24 years, nothing is known about the cutaneous receptors involved in gonopodium movement, or how the afferent signals are processed to determine the location of this organ during copulation. Using Western mosquitofish, *Gambusia affinis*, as our model, we attempt to fill this gap in knowledge. Preliminary data showed cutaneous nerves and sensory neurons innervating superficial neuromasts surrounding the base of adult male gonopodium; those cutaneous nerves projected ventrally from the spinal cord through the 14th dorsal root ganglion and its corresponding ventral root towards the base and fin rays of the gonopodium. We asked what role the cutaneous superficial neuromasts play in controlling the positioning and timing of the gonopodium’s fast and synchronous movements for effective sperm transfer. First, we found a greater number of superficial neuromasts surrounding the base of the male’s gonopodium compared to the base of the female’s anal fin. Second, we systemically removed superficial neuromasts surrounding the gonopodium base and observed significant impairment of the positioning and timing of gonopodial movements. Our findings provide a first step to supporting the following hypothesis: during radical reorganization of the Poeciliid body plan, superficial neuromasts have been partially co-opted as proprioceptors that allow the gonopodium to control precise positioning and timing during copulatory attempts.

## Introduction

In vertebrates, the tetrapod spinal motor system controlling paired appendages (i.e., forelimbs and hindlimbs) has proprioceptors (i.e., sensory inputs) that relay information regarding the length of skeletal muscles and the forces applied to them. The spinal motor system uses this information to ascertain variables, such as joint position, required to perform an array of movement(s) and innate behaviors (Mullins et al., 2011; Proske and Gandevia, 2012). For teleost fishes to move paired (i.e., pectoral and pelvic fins) and median unpaired appendages (i.e., dorsal and anal fins; located above and below the center of mass respectively), the spinal motor system controlling them must have a means of assessing a starting position and making postural adjustments to compensate for changes in the body’s center of mass as it shifts to different locations along the body axis (Lewis and Eisen, 2003; Murakami and Tanaka, 2011; Williams et al., 2013). For example, in the centrarchid fish, the bluegill sunfish, *Lepomis macrochirus*, nerve endings in the distal ends of pectoral fins appear to serve as proprioceptors that monitor pectoral fin rays as bluegill sunfish swim (Hale and Williams, 2012; Williams et al., 2013) or navigate through complex environments (Flammang and Lauder, 2013). However, the proprioceptors associated with median unpaired dorsal and anal fins of fishes in general are unknown.

For some teleost species, such as the live-bearing fish family Poeciliidae, unpaired fins have a role in addition to that of locomotion. Poeciliid fishes have internal fertilization, and male poeciliids possess a sexually dimorphic anal fin, the gonopodium, that functions as a copulatory organ to transfer sperm to the female reproductive tract. Most poeciliid species exhibit rapid copulation, likely with Western mosquitofish, *Gambusia affinis* (Baird and Girard, 1853; mosquitofish hereafter) being best studied in this regard. During mating attempts, a male mosquitofish approaches a female from a position largely outside the female’s field of view and circumducts the gonopodium; circumduction consists of four phases: abduction, adduction, extension-pronation, and torque-trust (Rivera-Rivera et al., 2010). The torque-trust sequence is the most crucial phase as the male performs a fast and synchronous maneuver to transfer encapsulated sperm bundles (spermatozeugmata) into the female urogenital sinus (genitalia) in <20 ms, regardless of their sexual receptivity (Rosa-Molinar, 2005; Rivera-Rivera et al., 2010). The mosquitofish efferent circuit that activates motor neurons innervating deep and superficial appendicular muscles of the gonopodium has been described (Rosa-Molinar, 2005; Rivera-Rivera et al., 2010; Serrano-Velez et al., 2014); however, the sensory receptors and the mechanisms that drive the male fast copulatory movement required for rapid copulation have not.

**Figure 1:**
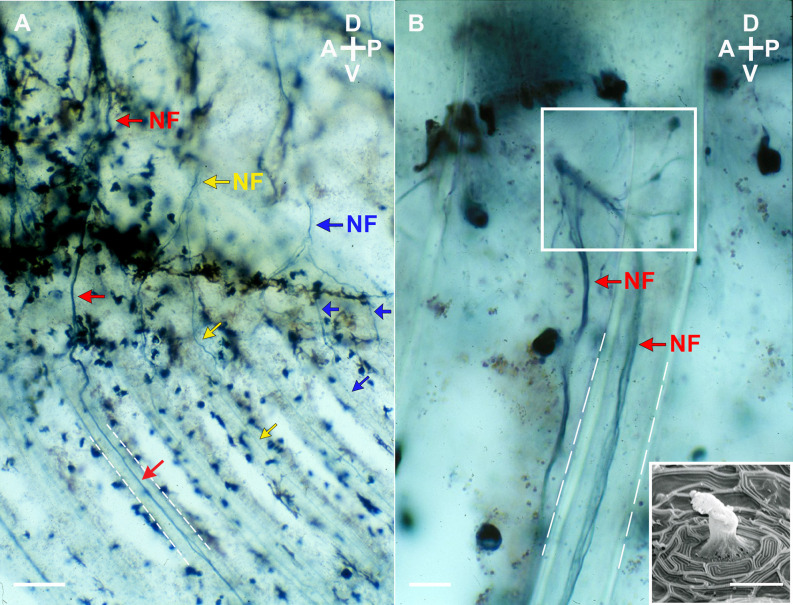
Lateral view (**A** and **B**) of nerve fibers projectingventrally from the 14th ventral root to the base and the fin rays (white dashed lines) of the gonopodium of an adult male mosquitofish. Nerve fibers (NF; red, yellow, and blue arrows as three representative fibers) were anterograde labeled with biotinylated dextran amine (BDA; Life Technologies, ThermoFisher Scientific, Grand Island, NY) and visualized with 3,3’,5,5’-Tetramethylbenzidine (TMB; Vector Laboratories, Burlingame, CA), a blue chromogenic substrate. Note in **(B)** that the BDA-labeled TMB visualized nerve fibers branch into superficial neuromasts clustered at the base of the gonopodium (see square). (Insert image in **B**) Scanning Electron Micrograph of a superficial neuromast from an adult male mosquitofish. Image in **(A)** and **(B)** were acquired on an Olympus Bright-field microscope (BH2 model) with an Olympus D Plan 10PO 10×/0.25 NA and an Olympus A 40 40×/0.65 NA objectives. Insert image in **(B)** was acquired on a cold field emission Scanning Electron Microscope (cfe-SEM; Hitachi High-Tech, S-4700-I series) with a secondary electron detector. Scale bars = 10 μm (**A** and the electron micrograph) and 50 μm **(B)**. A, anterior; P, posterior; D, dorsal; V, ventral.

In fishes and amphibians, superficial neuromasts have been shown to function as mechanosensory receptors located on the epithelium where the filament-like projections of the sensory cells, the hair cells, are directly exposed to water flow (Münz, 1989; Montgomery et al., 2000; Nagiel et al., 2008; Mogdans and Bleckmann, 2012). These receptors provide sensory inputs required for motor responses to detect changes in swimming direction and to recognize biotic or abiotic activity (Münz, 1989; Montgomery et al., 1995). Indeed, neuromasts are not restricted to the lateral line; they also can be found in the head and trunk regions of the fish and amphibian body. Even though studies of superficial neuromasts have focused strictly on their function in detecting motion in the surrounding water, these sensory structures might provide additional sensory inputs generated by fin movements (i.e., proprioception). However, superficial neuromasts’ functioning as proprioceptive receptors has not been demonstrated. In male mosquitofish, for example, the movement of the gonopodium at different angles will cause deformation of the skin, which can activate cutaneous afferents innervating this modified anal fin. Anterograde and retrograde labeling studies (unpublished data) revealed cutaneous nerve fibers projecting ventrally from the 14th ventral root of the spinal cord to the base and the fin rays of the adult male mosquitofish gonopodium (Figure 1A). These cutaneous afferent fibers branch into superficial neuromasts clustered at the base of the gonopodium (Figure 1B), but no nerve fibers were found between this cluster of superficial neuromasts and the lateral line. Instead, these cutaneous afferent fibers were found to connect in the spinal dorsal root ganglia (unpublished data). These findings support our reasoning on looking more closely at the superficial neuromasts surrounding the gonopodium and investigating whether these receptors play a role similar to proprioceptors for fin movement. Herein, we provide the first detailed assessment of the number and distribution of superficial neuromasts in adult female and male mosquitofish. Furthermore, we examine how the reorganization of these cutaneous receptors could control precise positioning and timing to perform fast and synchronous movements for copulatory behavior.

## Material and Methods

### Labeling superficial neuromasts

**Figure 2:**
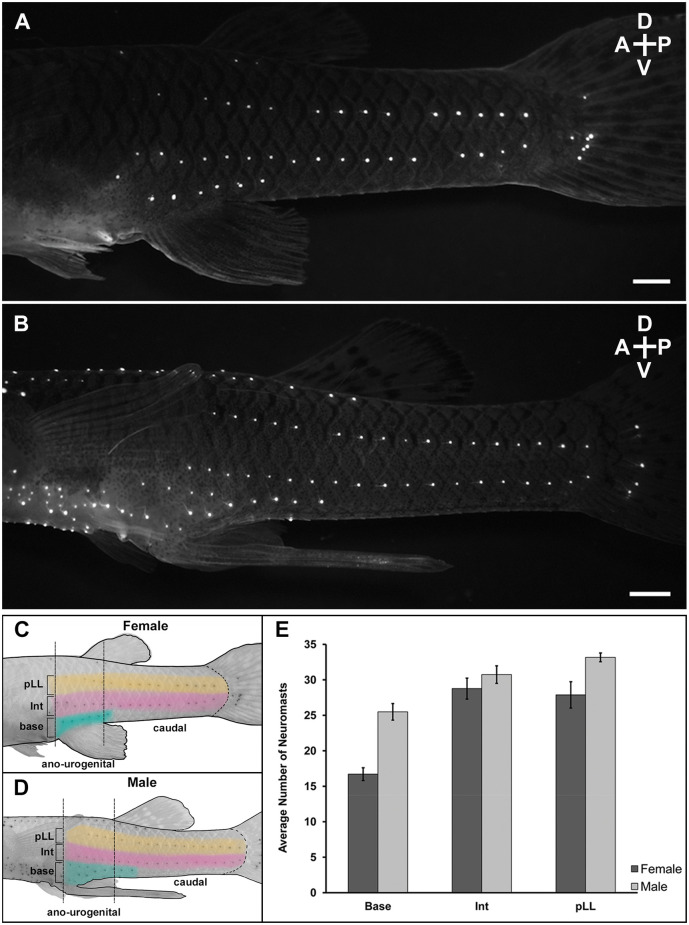
Lateral view of superficial neuromast labeling at the ano-urogenitaland caudal regions of mosquitofish. Adult female (*n* = 12) and adult male (*n* = 12) were immersed with 10 μM solution of the fluorescent dye 4-Di-2-asp (10 μM solution) dissolved in distilled water overnight at 20°C in a darkenedroom to minimize stress. The next day, the mosquitofish were washedsix times (5 min each) in clean water in the dark, anesthetized with Benzocaine diluted in water (1:2,000), placed in a shallow Petri dish with water and visualized using a Leica MZ-16 FA stereo fluorescence microscope with a 488 nm excitation filter. **(A)** 28 mm SL female and **(B)** 25 mm SL male mosquitofish. Scale bars = 10 μm. A, anterior; P, posterior; D, dorsal; V, ventral. Panels **(C)** and **(D)** are graphics of female and male mosquitofish showing the superficial neuromast labeling pattern and the three sections quantified within two anatomical regions (ano-urogenital and caudal) previously described for *G. affinis*; (base) base of the anal fin at the ano-urogenital region; (Int) intermedial; (pLL) posterior lateral line at the caudal region. Graphic design in **(C)** and **(D)** are inverted images from **(A)** and **(B)** respectively [created in Procreate^®^, 65% opacity, HEX #E1BE6A (yellow in pLL), CC79A7 (pink in Int), 40B0A6 [teal in base)]. Brightness and contrast were slightly adjusted for better display. **(E)** Average number of labeled superficial neuromasts from ano-urogenital, intermedial, and caudal regions after four repeated labeling. Base (*t* = 5.97, *df* = 21, *P* = 6.40 e-06), female (mean = 16.71, SD = 3.10); males (mean = 25.49, SD = 4.05). Intermediate (Int; *t* = 1.02, *df* = 21, *P* = 0.32), female (mean = 28.77, SD = 5.14); male (mean = 30.74, SD = 4.30). Posterior lateral line (pLL; *t* = 2.70, *df* = 13, *P* = 0.02), female (mean = 27.88, SD = 6.46), male (mean = 33.17, SD = 2.16). Female: *n* = 12, dark column; male: *n* = 12, light column. Black vertical bars = standard error.

**Figure 3:**
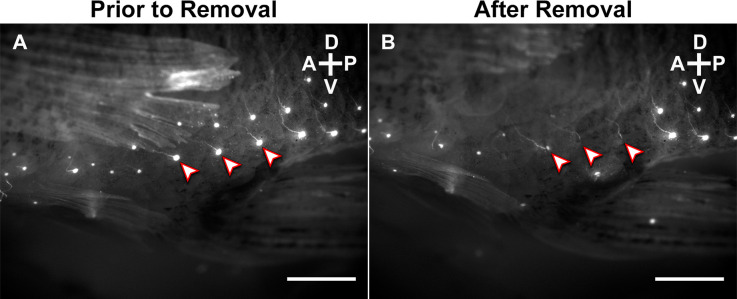
Lateral view of the base of the gonopodium of an adult male mosquitofish **(A)** before and **(B)** after removal of superficial neuromasts. Superficial neuromasts were stained with 10 μM solution of 4-Di-2-asp overnight at 20°C in a darkened room before 3D kinematics recording. “Arrowheads” highlight three representative neuromasts that were carefully removed using microsurgical forceps, preserving each superficial neuromast’s corresponding ascending nerve fibers. Scale bars = 20 μm. A, anterior; P, posterior; D, dorsal; V, ventral.

For this study, all *G. affinis* were obtained from a colony established at the University of Puerto Rico-Rio Piedras Animal Resources Facility, and all experimental procedures were approved by the Institutional Animal Care and Use Committee at this university. To visualize superficial neuromasts (Nakae et al., 2012), adult male (*n* = 12) and adult female (*n* = 12) mosquitofish (standard length: >20 mm) were labeled with the non-toxic fluorescent dye 4-(4-Diethylaminostyryl)-1-methylpyridinium iodide (4-Di-2-asp; 10 μM solution, Cat.# D3418, Sigma-Aldrich; Figures 2A,B, 3A,B).

### Visualization

After overnight immersion in the dye, mosquitofish were washed six times (5 min each) in clean water in the dark, anesthetized with a low dose of 4-Aminobenzoic acid ethyl ester, Ethyl 4-aminobenzoate (Benzocaine, 1:200 dilution, Cat.# E1501, Sigma-Aldrich) and placed in a fluorescence dissecting stereo microscope (Leica, MZ 16 FA) with a Green Fluorescent Protein filter set (~475 nm excitation, ~600 nm emission) for visualization. To quantify and analyze labeled neuromasts, a QImaging Retiga Exi camera and Qcapture Program (version 1.70.0) were used to acquire images from left to right lateral view images of the caudal ano-urogenital region (see Figure 2). After image acquisition, mosquitofish were placed in a crystal bowl with clean oxygenated water for 1 h to allow the fish recover from anesthesia; then they were returned to individual tanks in the animal care facility.

All adult male and female mosquitofish used for this visualization were chosen randomly from the colony, using the record of date of birth to confirm that all animals were at the beginning of adult stage. Adult mosquitofish can grow 2–4 mm standard length within 6–7 months. Hence, to assess whether the number and distribution of superficial neuromasts throughout the body remained consistent after 9 months, even if the mosquitofish grew, the entire procedure described above for labeling and visualization was repeated three additional times, 1 month following the first labeling, 2 months after the second labeling, and 5 months after the third labeling.

### Statistical analysis

Observations and quantification analyses of labeled superficial neuromasts in this study for both males and females were focused on two of three anatomical regions previously described in *Gambusia* (Rosa-Molinar et al., 1994, 1998; Rosa-Molinar, 2005): the ano-urogenital and caudal regions (see diagram in Figures 2C,D). In male Gambusia, the ano-genital region is specifically defined as the region between the 8th and 14th spinal vertebral segments where the 8th vertebra is aligned with the base of the anal fin (Rosa-Molinar et al., 1994, 1998; Rivera-Rivera et al., 2010; Langerhans and Rosa-Molinar, 2021 for better visual reference). Two-tailed *t*-tests were conducted to determine whether the sexes differed in the number of labeled superficial neuromasts. The temporal consistency of superficial neuromasts in individual fish was also evaluated and compared to determine if the number of superficial neuromasts within each region changed after 9 months.

### Labeling and removal of the superficial neuromasts

An independent experiment was conducted to assess the role of particular superficial neuromasts in copulatory behavior. Mating trials used “control” males with intact superficial neuromasts and “experimental” males that had superficial neuromasts removed. We first stained sexually experienced adult male mosquitofish (*n* = 20; standard length: >20 mm) using 4-Di-2-asp as previously described. For the experimental group, 1 day before the 3D kinematics recording and analysis was preformed, 10 males were anesthetized and, using microsurgical forceps and the MZ-16 FA stereo fluorescence microscope for visualization, all superficial neuromasts around the base of the gonopodium were carefully removed from both sides, preserving the corresponding ascending nerve fibers (Figures 3A,B). For the control group, 10 males were anesthetized as described for the experimental group, but no neuromasts were removed. Then, each sexually experienced adult male was placed in a crystal bowl with oxygenated water before beginning kinematics recording the next day.

### Recording and analysis of mating kinematics

Each experimental and control male mosquitofish was separately placed in a chamber with a sexually inexperienced but receptive adult female mosquitofish. From previous observations in the colony, sexually receptive females remain motionless in the water with their anal fin fully extended; this action pattern has been defined as standing. Sexually non-receptive females display a prominent sub-ocular bar while slightly bending the body, which is indicative of an aggressive stand. At all times, the chamber was maintained with well-oxygenated water and kept at standard water temperature (27°C–28°C). All recordings began immediately after the female mosquitofish was placed in the chamber. Each recording had a timeframe of 1 min. Based on previous video recording (e.g., Rivera-Rivera et al., 2010) and many observations of the colony, mating behavior in adult mosquitofish can occur in less than 1 min and repeate the same pattern: an adult male approaches and positions himself behind an adult female and circumducts the gonopodium fast enough to avoid female responses, such as swimming away or aggression. Mating kinematic recordings of fast copulatory attempts were performed twice a week for 1 month. Two criteria were considered for stopping the recording: (1) video over 1 min; or (2) increase in aggressive interactions due to restricted space.

**Figure 4:**
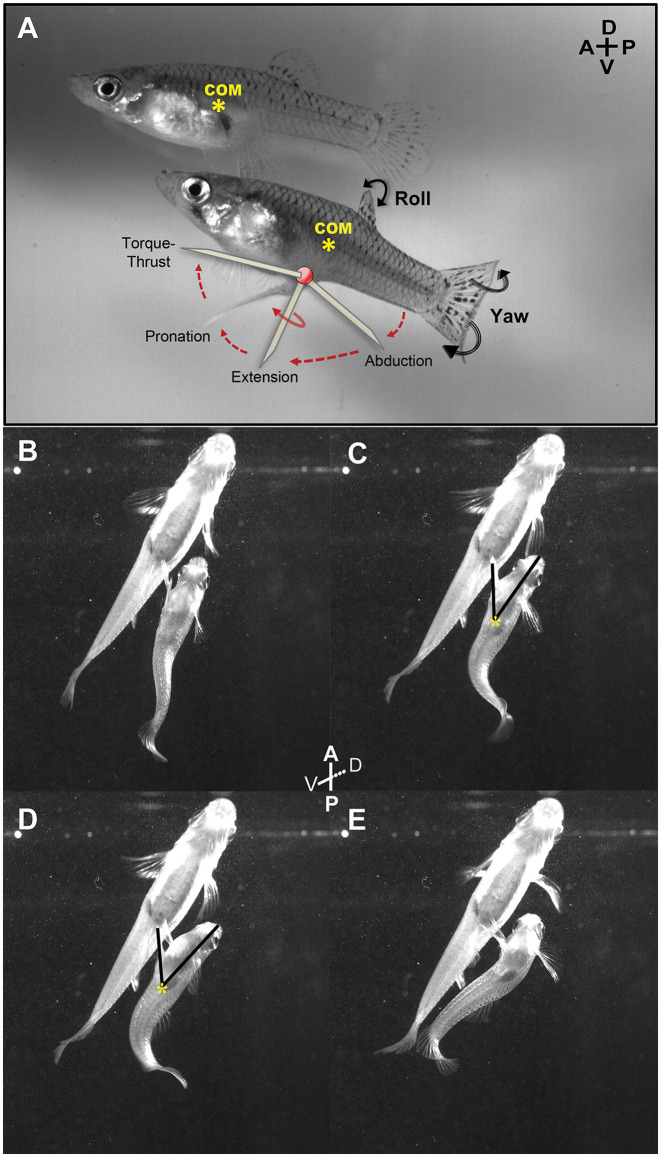
Lateral and ventral view of an adult male mosquitofish performing circumduction of the gonopodium. **(A)** Diagram shows all the gonopodial positions during circumduction (abduction, extension, rotation, and torque-thrust; each identified as diagonal lines). Center of mass (COM; yellow asterisk) was calculated in adult female mosquitofish to compare the localization with the COM in adult male mosquitofish. We observed the same localization of COM across all females recorded. Female COM was calculated anterior to the anal fin, very close to the gravid spot and the swim bladder, while male COM was more posteriorly positioned, dorsal to the gonopodium (see details in methods for analysis of mating kinematics). Ventral view of an adult male mosquitofish performing Torque **(B,C)** and Thrust **(D,E)** maneuvers of gonopodial circumduction with intact superficial neuromasts at the base of the gonopodium. Note that the tip of the gonopodium is in the closest position to the female genitalia during Thrust phase **(D)**, while the COM did not changeduring circumduction (yellow asterisk in **C** and **D**). Torque **(C)** and Thrust **(D)** phases of the circumduction were measured using the “angle tool” option within Image J 1.48i program; one ray segment was traced from the tip of the mouth to the COM (yellow asterisk) and the second ray segment was traced from the COM to the tip of the gonopodium. A, anterior; P, posterior; D, dorsal; V, ventral.

Three synchronized high-speed video cameras (two Photron Fastcams, and a Photron APX system) with 1,024 × 1,024 pixel resolution operating at 1,000•frames•s^−1^ (1/1,000•s shutter speed) and positioned to digitally record lateral, frontal, and ventral views of the chamber (Martínez-Rivera et al., 2010; Rivera-Rivera et al., 2010; Martínez-Rivera et al., 2014) recorded any copulatory attempts made by each male. In each mating attempt, males approached females from behind and circumducted their gonopodium while rapidly thrusting their body and gonopodium in an attempt to deposit sperm bundles in the female urogenital sinus (Bisazza, 1993; Rosa-Molinar, 2005; Plath et al., 2007; Rivera-Rivera et al., 2010). Four experimental males and seven control males performed the entire torque/thrust maneuvers of a copulatory attempt (Figure 4A).

We used Image J (version 1.48i) 1 software to calculate center of mass and angle of the gonopodium during both torque and thrust movements of circumduction (representative example in Figures 4B–E) in the 11 video sequences in which males completed the entire torque/thrust maneuver. Gonopodium angle provides an estimate of the magnitude of lateral rotation and extension of the gonopodium. We separately measured the gonopodium angle during torque and thrust positions because lateral rotation and full extension of the gonopodium during this last phase is critical for copulation. Likewise, males typically do not abort the circumduction attempt once engaged in this phase. In Image J, we measured the male body “area” and “center of mass” (COM) using the “analyze” tab in the ventral-view video sequences. We outlined the adult male mosquitofish body with the “freehand selection” tool; the software calculated the area of the mosquitofish body as well as the *x* and *y* coordinates for the COM (yellow asterisks in Figures 4C,D) estimated simply as the “average *x and y* position of selection”. To assess the angle of the gonopodium relative to the mid-line axis at each torque and thrust position, the “angle” tool option in Image J was used to trace one ray segment from the tip of the mouth to the COM point and the second segment from this centroid point to the tip of the gonopodium (Figures 4C,D). Moreover, we evaluated lateral-view video sequences to identify the relative center of mass along the anteroposterior axis for control and experimental adult male mosquitofish to observe whether the COM shifted along the body after removal of superficial neuromasts. In addition, we viewed front and lateral video sequences of all 20 males to count the frequency of four events: (1) the number of times male mosquitofish chased female mosquitofish; (2) the number of circumductions; and (3) the number of mating-attempt failures (i.e., males engaged in the entire copulation sequence but did not reach female’s urogenital sinus for sperm transfer). All high-speed video sequences (lateral, frontal, and ventral) were calibrated before any measurements were taken. For all experiments, all mosquitofish were observed for seven consecutive days following these procedures to assure that the anesthesia and forceps did not cause changes in innate behavior (i.e., swimming, eating) or inflict life-threatening injury. No changes in mosquitofish behavior were observed, and no skin scrapes, lesions, or infections were found. By 1 month, the superficial neuromasts that were removed had regenerated (endpoint of the recording). We continued monitoring the number and distribution of superficial neuromasts to make sure there was no aberrant increase in the number of superficial neuromasts that regenerated following their removal.

### Statistical analysis

We used two-tailed *t*-tests to determine whether gonopodium angle during either the torque phase or thrust phase differed between control and experimental males (intact and removed superficial neuromasts, respectively).

## Results

### Labeling, visualization, and quantification of superficial neuromasts

In all sampled adult male and adult female mosquitofish, superficial neuromasts were distributed in horizontal lines in the ano-urogenital and caudal regions, two of the three previously described regions of the novel body plan that consists of anterior trunk, posterior caudal, and ano-urogenital region (Rosa-Molinar et al., 1994, 1998). The horizontal lines of superficial neuromasts were found at the: (1) base of the anal fin (ano-urogenital region); (2) posterior lateral line (pLL; caudal region); and at (3) an intermediate (Int) location between the ano-urogenital and pLL (Figures 2A,B). In these three sections, the number of superficial neuromasts in adult male and adult female mosquitofish was compared. We found the sexes differed in the number of superficial neuromasts in the base of the anal fin/gonopodium (*t* = 5.97, *df* = 21, *P* < 0.0001) and the posterior lateral line (*t* = 2.70, *df* = 13, *P* = 0.02; Figure 2E). However, the number of superficial neuromasts in the intermediate section did not differ between males and females (*t* = 1.02, *df* = 21, *P* = 0.32; Figure 2E). In four repetitions of labeling, the distribution pattern, and the number of superficial neuromasts remained the same for every individual after 9 months.

### Kinematics show effect of removal of superficial neuromasts in adult males

**Figure 5:**
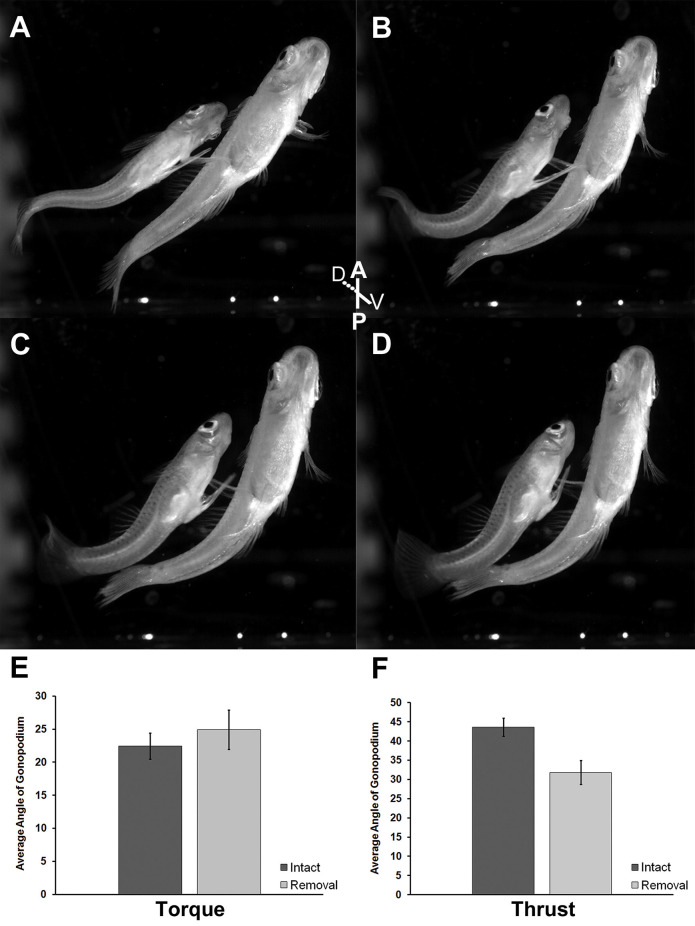
Ventral view of adult male mosquitofish performing Torque **(A,B)** and Thrust **(C,D)** maneuvers of gonopodial circumduction after removal of superficial neuromasts from the base of the anal fin. Average angle of gonopodium in the torque **(E)** and thrust **(F)** maneuvers of gonopodial circumduction after removal of superficial neuromasts. Torque (*t* = −0.72; *df* = 9; *P* = 0.49), control (*intact*; mean = 22.45, SD = 5.19; dark gray column), experimental (*removal*; mean = 24.91, SD = 5.91; light gray column). Thrust (*t* = 3.01; *df* = 9; *P* = 0.01), control (*intact*; mean = 43.59, SD = 6.22; dark gray column), experimental (*removal*; mean = 31.84, SD = 6.26; light gray column). Black vertical bars = standard error.

We found no effect of the removal of superficial neuromasts on gonopodium angle during the torque phase of circumduction (*t* = −0.72; *df* = 9; *P* = 0.49; Figures 5A,B,E). During the torque movement of the gonopodial circumduction, control males exhibited gonopodium angles that ranged from 16.16 to 32.99 degrees, while experimental adult males showed a similar range in angle from 17.28 to 31.71 degrees. However, control males exhibited a greater gonopodium angle during the thrust phase compared to experimental males (*t* = 3.01; *df* = 9; *P* = 0.01; Figures 5C,D,F). Before the thrust maneuver of control males, the angle of the gonopodium ranged from 36.89 to 53.40 degrees, while experimental males exhibited angles that ranged from 22.48 to 35.66 degrees.

Also, after evaluating lateral-view video sequences for the relative center of mass (COM) position along the anteroposterior axis in both control and experimental adult male mosquitofish, we observed no shift of COM along the body after removal of superficial neuromasts (Figure 4A). We further found that compared to experimental males, control males engaged in more female chases (15 vs. 10) and more circumduction and mating attempts (7 vs. 4), but had fewer failures to contact the female’s urogenital sinus [1 of 7 (14%) vs. 3 of 4 (75%)].

## Discussion

### Sex-specific distribution of superficial neuromasts

A first step in testing whether male poeciliid live-bearing fishes have evolved superficial neuromasts that function as mechanosensory and proprioceptive receptors critical for rapid copulation is to determine whether males and females differ in their neuromasts. Using *Gambusia affinis* as a model, we found sexual differences in the number but not the distribution of trunk superficial neuromasts. The largest difference was observed in the region near the sexually dimorphic anal fin, but differences also occurred in the posterior lateral line. The greater number of neuromasts in males might be associated with the anatomical transformation of the male body plan during sexual maturation (Rosa-Molinar et al., 1994, 1998; Rosa-Molinar, 2005). That is, in males the peripheral nervous system modification parallels skeletal and muscular changes required for effective gonopodial functioning during copulation to achieve reproductive success. However, to support the hypothesis regarding male/female differences in neuromast numbers, additional work must be undertaken to assess the number and distribution of superficial neuromasts of embryo and immature female and male mosquitofish, and whether similar results are observed in other poecilids at different life stages.

Similar distribution patterns of superficial neuromasts along the body have been described in other poeciliids, such as Trinidadian guppies (Fischer et al., 2013). Fischer et al. (2013) identified dorsal trunk, ventral trunk, and midventral line regions (posterior lateral line, intermediate and base of the anal fin respectively for mosquitofish), along with other regions. It is intriguing that no sex differences in the number of neuromasts were observed in guppies, but populations living under higher predation risk had more neuromasts in some regions (including midventral) than populations under lower predation risk. This suggests two things: (1) different species may show different patterns of sexual dimorphism in the number and function of neuromasts in particular regions, perhaps partially due to differences in mating behaviors; and (2) ecological factors such as predation should be considered as selective agents that may modify the distribution and/or number of superficial neuromasts in *G. affinis* and other poeciliids.

### Effect of removal of superficial neuromasts

Previous studies focusing on the lateral line system (Münz, 1989) have shown its function in recognition of water movements (Engelmann et al., 2000; Montgomery et al., 2003; Windsor and McHenry, 2009; Feitl et al., 2010), schooling (Faucher et al., 2010), and escape behavioral responses (Mirjany et al., 2011; Danos and Lauder, 2012). In female mosquitofish, mechanosensory receptors in all regions (posterior lateral line, intermediate and base of the anal fin) could respond to changes in the surrounding environment, such as male fish nearby. For example, in the mating recordings we observed females startled after male mosquitofish touched the urogenital sinus region, showed a fast escape response (i.e., C- and S-start-like movements) and swam away. In adult male mosquitofish, removal of superficial neuromasts at the base of the gonopodium appeared to impair performance of the torque-thrust movement of male copulatory behavior. The lower angle of the gonopodium during the thrust phase of experimental males may result from improper separation of the gonopodium from the body, and hinders contact with the urogenital sinus of adult female mosquitofish required for effective sperm transfer. Consistent with this notion, we found, on average, a dramatic reduction in successful copulations between control (87.5% success) and experimental males (25% success). Our results suggest the removal of these superficial neuromasts disrupts the kinematics of the gonopodium during copulation. Moreover, the data suggest the absence of these cutaneous afferents might disrupt sensory inputs required for this unpaired sexually dimorphic fin to sense some deformation of the skin, and therefore, its position in relation to the mosquitofish body. Thus, our results suggest an additional mechanosensory function for neuromasts associated with the base of the anal fin.

Removal of superficial neuromasts did not cause changes in male COM. Moreover, video sequences showed in circumduction attempts, fish locomotion and maneuvers were not affected, indicating that deprivation of these receptors does not alter swimming stability. It is possible the group of neuromasts located at the posterior lateral line detects water flow and provides inputs for swimming and maneuvers, as previously reported (Münz, 1989; Engelmann et al., 2000; Montgomery et al., 2003; Windsor and McHenry, 2009; Feitl et al., 2010). Thus, it is more likely the propensity for chasing females would not be affected.

In summary, although removal of superficial neuromasts in the adult male mosquitofish does not affect COM or swimming maneuvers, removal appears to disrupt the mechanical functionality of the gonopodium. Thus, superficial neuromasts associated with the sexually dimorphic fin, the gonopodium, may serve as proprioceptors.

### Superficial neuromasts as proprioceptors

The perception of movement and sense of position, proprioception, of vertebrate limbs such as the paired and unpaired (i.e., dorsal, anal, and caudal) fins of fishes are critical for voluntary movement(s) and innate behaviors (Ono, 1979). It is well known that the coordination and synchronization of fins and body motion are important motor responses in fish balance, swimming, and propulsion (Webb, 1988, 2006; Blake, 2004). Although studies do not extensively discuss fish perception, proprioceptors serve as sensors to provide information such as joint angle, muscle length, and muscle tension. This information is integrated into the central nervous system, giving fish a sense of the relative position of the fin to the body and the effort employed in movement (Ono, 1979; Webb, 1988; Lewis and Eisen, 2003). In teleost fishes, peripheral sensory components are generally in the skin and/or surrounding the base of fins (Whitear, 1952, 1971; Nakae et al., 2012), which may result in physiological differences depending on the sensory nerve involved. For example, free nerve endings in vertebrates extend from the connective tissue (dermis) into the epithelial layers, and they respond mostly to tactile, thermal, and pain senses. The afferent innervation of these nerve endings is dominated by Aδ fibers, while other cutaneous receptors are dominated by Aβ fibers. Until recently, free nerve endings in fish were associated with tactile and pain receptors, not proprioceptors (Whitear, 1971). Williams et al. (2013) reported immunolabeling and electrophysiological activities of afferent nerve fibers innervating pectoral fins of adult bluegills, suggesting that these nerve fibers could provide proprioceptive inputs of fin position in space. Williams et al. (2013) also suggested that, in addition to being important to other biotic interactions, sense of fin position may be an important element during copulation. However, until now the location of the proprioceptive-like receptors involved in proprioception of fin movement was unknown.

Mechanosensory receptors have been extensively described and proven to respond to vibration, pressure, touch, and tension from the environment. There is no doubt that superficial neuromasts of the lateral line and caudal region provide such inputs to the central nervous system in fishes. However, it could be expected that if all superficial neuromasts have the same function, then removal of neuromasts at the base of the gonopodium would not significantly affect the maneuvers of circumduction and copulatory attempts since the remaining intact neuromasts close to the gonopodium, as well as in the lateral line and caudal region, provide the same inputs of water motion to the fish. Thus, superficial neuromasts at the base of the gonopodium appear to have a unique sensory innervation associated to the skin deformation which could be connected to afferent projections innervating the “joints” between proximal pterygiophores (interhaemal bones) and anal fin lepidotrichia (fin spines and rays).

Major evolutionary transitions, such as changes in body plan, invading novel environments, and evolution of novel traits, can cause rapid adaptive diversification and lead to new evolutionary routes because evolutionary change is contingent on past evolutionary history (Gould, 2002; McLennan, 2008; Losos, 2009; Wellborn and Langerhans, 2015). Subsequent to the evolution of the novel three-part body plan of poeciliid fishes, recent work has suggested that traits within the gonopodial complex have been co-opted for new evolutionary functions (Langerhans and Rosa-Molinar, 2021; and references therein). Similarly, we propose that Poeciliids may have remodeled or co-opted some sensory structures to adapt to changes in the novel ano-urogenital region and associated mating behaviors to fulfill the new copulatory function. That is, superficial neuromasts were previously described as mechanosensory cells providing inputs of surrounding water movements; however, for male mosquitofish specifically, these superficial neuromasts at the base of the gonopodium can have an additional role in providing inputs of the gonopodial position and movements during the maneuvers of circumduction and mating attempts as the skin bends. For this reason, we hypothesize that during radical reorganization of the Poeciliid body plan, superficial neuromasts have been partially co-opted as proprioceptive receptors that allow for precise control of gonopodial positioning and timing during copulatory attempts, regardless of courtship display or female sexual receptivity. This hypothesis points to the need for more investigations to discover how superficial neuromasts may have been partially co-opted as proprioceptive receptors and how they may be integrated into a “central body map” in male Poeciliid fishes. For this, molecular structures, mechanisms, and pathways associated with these specific receptors are crucial to explore. Moreover, we need to discover the evolutionary links between these partially co-opted sensory structures and developmental, genetic, morphological, and behavioral traits within the Poeciliid family.

## Data Availability Statement

The raw data supporting the conclusions of this article will be made available by the authors, without undue reservation.

## Ethics Statement

The animal study was reviewed and approved by Institutional Animal Care and Use Committee at University of Puerto Rico-Rio Piedras campus, San Juan, Puerto Rico.

## Author Contributions

NM-R and IT-V developed the fish labeling protocol and experiments. JS-V, NM-R, and ER-M performed video recording and conducted image acquisition. NM-R and ER-M conducted image analyses. IT-V conducted double-blind counting of labeled superficial neuromasts. The manuscript was written by NM-R with editing help from JS-V, IT-V, RL, and ER-M. ER-M performed all the neural tract tracing. All authors contributed to developing research questions, strategies, and discussions. All authors contributed to the article and approved the submitted version.

## Funding

Research is partially supported by grants to ER-M from NIH (NS-39405), NSF (1002410), National Institute of General Medical Sciences (NIGMS-115042), National Institute of Mental Health (106245), and Puerto Rico Science, Technology and Research Trust (ER-M Agreement 2013-000034). ER-M is partially supported, and NM-R is fully supported by an NSF grant (NSF/HRD-1137725) to the Puerto Rico Center for Environmental Neuroscience (PRCEN), a Center for Research Excellence in Science and Technology (CREST).

## Conflict of Interest

The authors declare that the research was conducted in the absence of any commercial or financial relationships that could be construed as a potential conflict of interest.

## Publisher’s Note

All claims expressed in this article are solely those of the authors and do not necessarily represent those of their affiliated organizations, or those of the publisher, the editors and the reviewers. Any product that may be evaluated in this article, or claim that may be made by its manufacturer, is not guaranteed or endorsed by the publisher.
